# Molecular and morphological investigations on the renal mechanisms enabling euryhalinity of red stingray *Hemitrygon akajei*

**DOI:** 10.3389/fphys.2022.953665

**Published:** 2022-08-09

**Authors:** Naotaka Aburatani, Wataru Takagi, Marty Kwok-Shing Wong, Shigehiro Kuraku, Chiharu Tanegashima, Mitsutaka Kadota, Kazuhiro Saito, Waichiro Godo, Tatsuya Sakamoto, Susumu Hyodo

**Affiliations:** ^1^ Atmosphere and Ocean Research Institute, The University of Tokyo, Kashiwa, Japan; ^2^ Department of Biomolecular Science, Toho University, Funabashi, Japan; ^3^ Department of Genomics and Evolutionary Biology, National Institute of Genetics, Mishima, Japan; ^4^ Department of Genetics, Sokendai (Graduate University for Advanced Studies), Mishima, Japan; ^5^ Laboratory for Phyloinformatics, RIKEN Center for Biosystems Dynamics Research, Kobe, Japan; ^6^ Ushimado Marine Institute, Faculty of Science, Okayama University, Setouchi, Japan

**Keywords:** cartilaginous fish, euryhalinity, osmoregulation, urine, nephron, membrane transporter, FW adaptation, Batoidea

## Abstract

Most cartilaginous fishes live in seawater (SW), but a few exceptional elasmobranchs (sharks and rays) are euryhaline and can acclimate to freshwater (FW) environments. The plasma of elasmobranchs is high in NaCl and urea concentrations, which constrains osmotic water loss. However, these euryhaline elasmobranchs maintain high levels of plasma NaCl and urea even when acclimating to low salinity, resulting in a strong osmotic gradient from external environment to body fluid. The kidney consequently produces a large volume of dilute urine to cope with the water influx. In the present study, we investigated the molecular mechanisms of dilute urine production in the kidney of Japanese red stingray, *Hemitrygon akajei*, transferred from SW to low-salinity environments. We showed that red stingray maintained high plasma NaCl and urea levels by reabsorbing more osmolytes in the kidney when transferred to low salinity. RNA-seq and qPCR analyses were conducted to identify genes involved in NaCl and urea reabsorption under the low-salinity conditions, and the upregulated gene expressions of Na^+^-K^+^-Cl^-^ cotransporter 2 (*nkcc2*) and Na^+^/K^+^-ATPase (*nka*) were found in the FW-acclimated individuals. These upregulations occurred in the early distal tubule (EDT) in the bundle zone of the kidney, which coils around the proximal and collecting tubules to form the highly convoluted structure of batoid nephron. Considering the previously proposed model for urea reabsorption, the upregulation of *nkcc2* and *nka* not only causes the reabsorption of NaCl in the EDT, but potentially also supports enhanced urea reabsorption and eventually the production of dilute urine in FW-acclimated individuals. We propose advantageous characteristics of the batoid-type nephron that facilitate acclimation to a wide range of salinities, which might have allowed the batoids to expand their habitats.

## Introduction

The class Chondrichthyes, commonly known as cartilaginous fishes, currently contains over 1,250 living species (Ebert and Dando, 2020), and more than 90% of these are obligate marine species (Ballantyne and Robinson, 2010). Their osmoregulatory strategy is characterized by the presence of high concentrations of NaCl and urea in the body fluid to cope with the high external osmolality in marine environment (ureosmotic strategy) (Payan et al., 1973; Pang et al., 1977). The high levels of urea (300–450 mM) are essential for maintaining their body fluid iso- or slightly hyperosmotic to the surrounding seawater (SW) (Ballantyne and Fraser, 2012), thereby avoiding dehydration (Greenwell et al., 2003; Anderson et al., 2007). Meanwhile, a limited number of elasmobranch (the sharks, rays, and skates) species can tolerate low-salinity water and are known as euryhaline elasmobranchs. Indeed, euryhaline elasmobranchs, such as bull shark *Carcharhinus leucas* (Thorson, 1971), Atlantic stingray *Hypanus sabinus* (Piermarini and Evans, 1998), and largetooth sawfish *Pristis microdon* (Smith, 1931), have been reported to inhabit freshwater (FW) environments during parts of their life cycles. The most distinctive physiological feature of euryhaline elasmobranchs is that they retain relatively high internal NaCl and urea levels (osmolality >600 mOsm) in FW environment (Hazon et al., 2003), which is approximately twice the plasma osmolalities of FW teleosts (Ballantyne and Fraser, 2012). FW-acclimated euryhaline elasmobranchs thus face a steep inwardly directed osmotic gradient between internal and external environments.

Several previous studies suggest that the gill, rectal gland, and kidney contribute to maintaining the high internal NaCl level in the low-salinity environment. In the case of gills, elasmobranchs have two types of branchial ionocytes and the number of these cells increased in FW-acclimated Atlantic stingray (Piermarini and Evans, 2001; Choe et al., 2005) and 30% SW-acclimated Japanese banded houndshark *Triakis scyllium* (Takabe et al., 2016). Furthermore, branchial mRNA levels of Na^+^/K^+^-ATPase (*nka*) and Na^+^/H^+^ exchanger type-3 (*nhe3*) were significantly increased following the acclimation to low-salinity environment in bull shark (Reilly et al., 2011), Atlantic stingray (Choe et al., 2005), and houndshark (Takabe et al., 2016). These findings suggest the potential contribution of branchial ionocytes in euryhaline elasmobranchs to NaCl uptake in low-salinity environments. Rectal gland is another osmoregulatory organ dedicated to the excretion of excess NaCl in marine environment. The size and NKA activity of rectal gland decreased following the FW-acclimation in bull shark (Oguri, 1964; Pillans et al., 2005) and Atlantic stingray (Piermarini and Evans, 2000), implying that the excretion of NaCl from the rectal gland is suppressed in low-salinity environments to maintain the high internal NaCl level. Meanwhile, kidney is the only organ able to excrete excess water from the body with concomitant retention of ions and urea (Olson, 1999). It is well known that the kidney of teleost fishes produces large amounts of dilute urine to overcome overhydration in the hypo-osmotic environments (Edwards and Marshall, 2012). In euryhaline Atlantic stingrays, both the glomerular filtration rate (GFR) and urine flow rate (UFR) were markedly increased after transfer from ambient SW to 50%-diluted SW, suggesting that the kidney of stingrays vigorously excretes excess water gained from the diluted environment as in the case of FW teleost fishes (Janech et al., 2006). The concomitant increases in reabsorption of NaCl and urea to produce dilute urine in the hypo-osmotic environment were also demonstrated in the Atlantic stingray (Janech et al., 2006) and bull shark (Imaseki et al., 2019). In bull shark, Na^+^-Cl^-^ cotransporter (NCC) was suggested to be a key renal transporter facilitating the reabsorption of both NaCl and urea in the FW-acclimated bull shark (Imaseki et al., 2019). However, whether the molecular mechanism to produce dilute urine is shared between bull shark and other euryhaline elasmobranch species remains unknown.

Recently, we reported that the kidney of Japanese red stingray *Hemitrygon akajei* has a higher capacity to reabsorb NaCl and urea than that of cloudy catshark *Scyliorhinus torazame* in SW. The difference in osmolyte compositions of the plasma and urine between stingray and catshark was supported by previous data obtained in little skate *Leucoraja erinacea* (Stolte et al., 1977), whitespotted bamboo shark *Chiloscyllium plagiosum* (Wong and Chan, 1977), spiny dogfish *Squalus acanthias* (Thurau et al., 1969), and bull shark (Imaseki et al., 2019), suggesting that the kidneys of batoids (rays and skates) have higher capacity to reabsorb NaCl and urea than those of selachians (sharks) (Aburatani et al., 2020). The enhanced renal reabsorption is likely related to the anatomical characteristics of the batoid nephrons, where the early distal tubule (EDT) is highly convoluted and coiled around the proximal and collecting tubules (Aburatani et al., 2020). The higher capacity to reabsorb NaCl and urea is also advantageous for acclimation to low-salinity environments. In fact, several phylogenetically diverse species of batoids including largetooth sawfish, Atlantic stingray, and giant freshwater stingray *Urogymnus polylepis* are euryhaline (Last et al., 2016; Grant et al., 2019), whereas only few species of the family Carcharhinidae are known to be euryhaline among selachians (Ballantyne and Fraser, 2012). These facts indicate that euryhaline batoids have distinct characteristics from bull shark in renal mechanisms for adaptation to low-salinity environment.

Here, we performed transfer experiments of red stingrays from SW to low-salinity environments and conducted a comprehensive search for differentially expressed genes (DEGs) by RNA sequencing (RNA-seq). The mechanism to produce dilute urine was examined by integrating transcriptomic, physiological, and histochemical approaches. This new knowledge of renal mechanisms in euryhaline batoids will further improve our understanding of how elasmobranchs acquired euryhalinity during their evolutionary history.

## Materials and methods

### Animals

Male and female red stingrays, *Hemitrygon akajei* (Muller & Henle, 1841), were caught in a bay at Ushimado and transported to the Atmosphere and Ocean Research Institute (AORI), The University of Tokyo. One or two stingrays were kept in a 500-L experimental tank filled with recirculating natural SW (35–36‰) at 20°C under a constant photoperiod (12 L: 12 D) without feeding. Stingrays of both sexes that were caught in January, May, and November 2018 (average disc width = 33.6 ± 2.6 cm, average body weight = 1.5 ± 0.4 kg; *N* = 13) were used for a transfer experiment from SW to FW (Experiment 1). For urine collection (Experiment 2), only female stingrays caught in November and December 2021 and February 2022 (average disc width = 49.7 ± 3.3 cm, average body weight = 4.6 ± 0.6 kg; *N* = 11) were used to avoid possible contamination of seminal fluid into the urine sample in males (Kempton, 1953). All procedures for animal experiments were approved by the Animal Ethics Committee of Atmosphere and Ocean Research Institute of The University of Tokyo (P19-2). The present study was carried out in compliance with the ARRIVE guidelines.

### Transfer experiment 1: Tissue sampling for the analysis of gene expressions

Before the transfer experiment, stingrays were accommodated in the experimental tanks for at least 3 days in order to acclimate to the new environments. During the first 3 days of experiment, the salinity of the experimental tank water was reduced by 10–20% per day by replacement with FW to achieve a salinity of approximately 50% SW on day 3. The replaced FW was dechlorinated at the same temperature as the experimental tanks. The salinity was held for 4 days (day 4–7) to allow the stingrays acclimating to a brackish environment. Then, from day 8–11, the salinity of the tank water was further lowered 10–15% per day and reached nearly FW condition (osmolality, less than 25 mOsm/kg; Cl^−^: less than 13 mM) on day 11. Our preliminary experiment suggested that the two-step dilution protocol is suitable for successful acclimation of stingrays to FW environment without mortality. In this transfer regime, the stingrays were exposed to hypo-osmotic conditions for at least 7 days (after day 3). On day 11, more than 6 h after reaching a FW-acclimated condition, the stingrays were euthanized with 0.02% (w/v) ethyl 3-aminobenzoate methanesulfonate (Sigma-Aldrich, MO, United States) buffered with an equal amount of sodium bicarbonate (Sigma-Aldrich). Blood samples were obtained from tail vasculature using a syringe and 22G needle and were centrifuged at 10,000 × *g* for 10 min at 4°C to obtain the plasma. Plasma samples were stored at −30°C or −80°C until use. The whole kidney was dissected out and separated into the left and right halves. One half was immediately frozen with liquid nitrogen and stored at -80°C until RNA extraction, while the other half was fixed in modified Bouin’s solution (saturated picric acid: formalin = 3: 1) for 2 days at 4°C and then preserved in 70% ethanol at 4°C until use. The SW control individuals were kept in SW without dilution for 14 days in the experimental tank.

Cations (sodium, calcium, and magnesium) concentrations of holding water and plasma were measured with an atomic absorption spectrophotometer (Z5300, Hitachi, Tokyo, Japan). Chloride ion concentration was measured by a digital chloridometer (C-50AP, Jokoh, Japan). Osmolality was measured by a vapor pressure osmometer (5,600, Wescor, UT, United States). Urea concentration was measured by a colorimetric method according to Rahmatullah and Boyde (1980).

### Transfer experiment 2: Urine collection from SW control and 5% SW-acclimated stingrays

To collect urine from conscious individuals, the following surgical procedure was conducted during the transfer experiment. To lower the osmotic stress that may add to the surgical stress, the final dilution of environmental water was stopped at 5% SW instead of FW. After the initial 3 days of the acclimation period, stingrays were transferred to a low-salinity environment as described in experiment 1, except that replacement of FW was stopped at 5% SW (osmolality, 53.4 ± 3.7 mOsm/kg; Cl^−^, 26.0 ± 2.1 mM). After reaching to 5% SW, stingrays were anesthetized and blood samples were obtained from tail vasculature using a syringe and 22G needle with 0.2% (w/v) potassium EDTA as an anticoagulant. Blood samples were centrifuged at 10,000 × *g* for 10 min at 4°C to obtain the plasma. A polyethylene cannula (SP-45 or SP-55, Natsume Seisakusyo, Tokyo, Japan) was inserted into the protruded opening of urinary tract and was tied to the surrounding tissue by surgical suture. The other side of cannula was connected and secured to a 2-ml sampling tube where excreted urine was collected into (see also Aburatani et al., 2020). Stingrays were recovered from anesthesia by irrigating the gills with aerated holding water and then returned to a 100L-container tank (700 cm × 500 cm × 410 cm) for urine collection. Excreted urine was collected from conscious animals up to 48 h after the blood sampling. Plasma and urine samples were stored at −30°C until use. Ions, urea, and osmolality of the holding water, plasma, and urine were measured as described above. To estimate the reabsorption rates of osmolytes, we calculated solute reabsorption rates of major osmolytes by borrowing the reported values for glomerular filtration rate (GFR) and urine flow rate (UFR) in Atlantic stingray (Janech et al., 2006). The average values of the Atlantic stingray reared in harbor water were adopted for the calculation in the SW control individuals, while those in 50% diluted harbor water were adopted for the calculation in the 5% SW-acclimated red stingray. The solute reabsorption rates were calculated using (P_osm_ × GFR)–(U_osm_ × UFR) where P_osm_ and U_osm_ are concentrations of the osmolytes in plasma and urine, respectively.

### RNA-seq analysis

Total RNA was extracted from the frozen kidney with ISOGEN (Nippon Gene, Toyama, Japan) and treated with DNase I to digest genomic DNA. Purification was conducted using the Zymo RNA Clean & Concentrator Column (Zymo Research, CA, United States). The quantity and quality of the RNA were examined with the Qubit RNA HS Assay Kit on a Qubit 2.0 Fluorometer (Thermo Fisher Scientific, MA, United States) and the RNA 6000 Nano Kit on a 2,100 Bioanalyzer (Agilent Technologies, CA, United States). Sequencing libraries were prepared using 1 µg of the RNA with the TruSeq Stranded mRNA Sample Prep Kit (Illumina, CA, United States) and the TruSeq single-index adaptor (Illumina). The libraries were sequenced with HiSeq1500 (Illumina), and single reads of 80 bases were obtained. The adaptor sequences and low-quality reads were trimmed with Trim Galore! v0.4.0 (https://www.bioinformatics.babraham.ac.uk/projects/trim_galore/), and sequence quality was checked by fastq quality filter v0.11.3 (https://www.bioinformatics.babraham.ac.uk/projects/fastqc/). The trimmed reads were then assembled *de novo* using Trinity v2.3.2 (Grabherr et al., 2011) to obtain transcript contigs. The contigs were annotated using ncbi-blast-2.6.0+ (McGinnis and Madden, 2004). The trimmed reads were mapped to the assembled contigs using bowtie2 v2.3.0 (Langmead and Salzberg, 2012), and the expressions were quantified with eXpress v1.5.1 (Cappé and Moulines, 2009). The differential expression analysis was then conducted by edgeR v3.0.0 (Robinson et al., 2010; McCarthy et al., 2012). The raw sequenced reads were deposited in the DNA Data Bank of Japan (DDBJ) under the accession numbers DRX363233, DRX363234, DRX363235, DRX363236, DRX363237, and DRX363238.

To investigate candidate gene contributing to FW acclimation, contigs annotated as genes known for ions and urea transport were shortlisted. Amino acid sequences of human *Homo sapiens* and western clawed frog *Xenopus tropicalis* were used as a query for local TBLASTN search, and the sequences of top-hit contigs were sent to NCBI BLASTX to obtain an annotation. When different contigs were annotated as the same protein or when the contig was annotated as a “hypothetical protein,” their orthologies were checked by inferring gene trees based on deduced amino acid sequences of the contigs using a web tool; ORTHOSCOPE (Inoue and Satoh, 2019; http://yurai.aori.u-tokyo.ac.jp/orthoscope/Vertebrata.html)

### cDNA cloning and RNA probe synthesis

Total RNA was extracted from the frozen kidney as described above. Complementary DNA was synthesized using High-Capacity cDNA Reverse Transcription Kit (Thermo Fisher Scientific) from 2 μg of total RNA pretreated with TURBO DNase-free kit (Thermo Fisher Scientific). Primer sets were designed to amplify cDNAs encoding NCC (858 bp; Genbank accession No. LC706818), elongation factor 1α1 subunit (EF1α1, 847 bp; Genbank accession No. LC706817), and β-actin (ACTB, 850 bp; Genbank accession No. LC715734) based on the contig sequence data from the transcriptome database. PCR was performed using KAPA Taq Extra (Kapa Biosystems, MA, United States) with the kidney cDNA as templates. The amplified products were ligated into pGEM-T Easy Vector (Promega, WI, United States) and transformed into *Escherichia coli* XL1-Blue cells. Plasmids were prepared from positive bacterial culture using FastGene Plasmid Mini Kit (NIPPON Genetics Co., Ltd., Tokyo, Japan) according to the manufacturer’s protocol. The cloned cDNAs were sequenced using BigDye Terminator v3.1 Cycle Sequencing Kit (Thermo Fisher Scientific) and an automated DNA sequencer (ABI PRISM 3100, Applied Biosystems, CA, United States). Primer sets used in this study are shown in Supplementary Table S1.

To synthesize a digoxigenin (DIG)-labeled RNA probe, the insert region of the sequenced plasmid was amplified with Primestar GXL (Takara Bio, Shiga, Japan) using the vector-specific M13 Forward and Reverse primers and subsequently purified with Wizard SV Gel and PCR Clean-up System (Promega). The purified DNA contains T7 and SP6 promotor sequences flanking the insert. Antisense and sense RNA probes were then synthesized from the purified DNA fragments using DIG RNA Labeling Kit (Roche Applied Science, Mannheim, Germany) with either T7 or SP6 RNA polymerase according to the manufacturer’s protocols.

### Quantitative real-time PCR

The gene expression levels were measured by quantitative real-time PCR (qPCR) using 7900HT Fast Real Time PCR System (Applied Biosystems) with a KAPA SYBR FAST qPCR Kit (Kapa Biosystems). The plasmids containing target sequences were serially diluted as standard templates for quantification in qPCR assay. The copy numbers were calculated using Sequence Detection System software v2.4 (Applied Biosystems). To explore the suitable gene for internal control in qPCR assay, the mRNA expression levels of β-actin (*actb*) and elongation factor 1α1 (*ef1α1*) were compared (Supplementary Figure S1). Since *ef1α1* mRNA expression was more stable between SW control and FW-acclimated individuals than that of *actb*, we adopted *ef1α1* as the reference gene for normalization. Primer sets for qPCR assay were designed using PrimerQuest (https://www.idtdna.com/Primerquest/Home/Index). Six individuals (three males and three females) were used for both the SW control and FW transfer.

### *In situ* hybridization

The fixed kidney was dehydrated with a series of ethanol, cleared in methyl benzoate, and embedded in Paraplast (McCormick Scientific, IL, United States). Serial sections were made at 7 μm thickness. Eight sections (four from SW control and four from FW-acclimated stingrays) were mounted onto a single MAS-GP-coated slide (Matsunami Glass, Osaka, Japan) for comparison of the signal intensities between SW and FW individuals with the same tissue section processing. Deparaffinized sections were treated with 2.5 μg/ml proteinase K (Sigma-Aldrich) and then hybridized with DIG-labeled RNA probes in hybridization buffer (50% formamide, 5 × SSC, 40 μg/ml bovine calf thymus DNA) at 58°C for 2 days. After hybridization, sections were serially washed in 2 × SSC for 30 min at room temperature, 2 × SSC for 1 h at 65°C, and 0.1 × SSC for 1 h at 65°C. The hybridized RNA probes were detected using Anti-Digoxigenin-AP, Fab fragment (1:5,000, Roche Applied Science). Hybridization signals were visualized with 4-nitro blue tetrazolium chloride and X-phosphate/5-bromo-4-chloro-3-indolyl-phosphate. Sections were counterstained with Kernechtrot Stain Solution (MUTO PURE CHEMICALS, Tokyo, Japan) and mounted using Permount (Fisher Chemical, NJ, United States). For image analysis described below, stained sections were mounted using CC/Mount (Diagnostic BioSystems, CA, United States) without counterstaining. Micrographs were obtained using a digital camera (DXM1200; Nikon, Tokyo, Japan).

### Image analysis

Image analysis was performed to examine the signal intensity among nephron segments. The micrographs were captured using identical conditions and analyzed with ImageJ package Fiji (Schindelin et al., 2012). Basal and apical membranes were designated using the polygon selection tool. Areas and total signal intensities were measured between the basal and apical membranes to calculate the mean tubular intensity/area. Three randomly selected images were examined for each individual to consider variations among tubules.

### Statistical analysis

Values were expressed as means ± s. e.m. After the assumption of normality with Shapiro–Wilk test, data were compared using two-tailed Student’s or Welch’s *t*-test according to whether the covariance were found or not. When the values were not normally distributed, the Mann–Whitney *U*-test was used instead. *p* < 0.05 was considered as statistically significant. Holm method was applied to the calculated *p* values for multiple comparisons. Statistical analysis was performed using Kyplot 6.0 software (Kyenslab, Tokyo, Japan).

## Results

### Changes in plasma and urine compositions following the acclimation to low-salinity environments

Tables 1, 2 show the osmolality and ion concentrations of plasma and urine in experiments 1 and 2, respectively. In SW control individuals, the plasma was composed of high levels of NaCl and urea, which contribute to an osmolality that was nearly identical to the holding SW (1,012.8 ± 5.4 mOsm/kg in experiment 1 and 1,054.5 ± 5.4 mOsm/kg in experiment 2). All plasma parameters, except for Ca^2+^ and Mg^2+^ in experiment 2, were significantly decreased after acclimation to the low-salinity environments. However, considerable concentrations of Na^+^, Cl^−^, and urea were maintained in the plasma even in low-salinity environments, resulting in a steep osmotic gradient between the plasma and surrounding environment (for plasma, 605.7 ± 8.0 mOsm/kg in experiment 1 and 623.5 ± 20.7 mOsm/kg in experiment 2; for environmental water, 17.0 ± 4.0 mOsm/kg in experiment 1 and 53.4 ± 3.7 in experiment 2, respectively).

**TABLE 1:** Plasma compositions of red stingray in Experiment 1

	N	Osmolality (mOsm/kg)	Na^+^ (mM)	Ca^2+^ (mM)	Mg^2+^ (mM)	Cl^−^ (mM)	Urea (mM)
Plasma							
SW	6	1,042.2 ± 6.7	329.8 ± 5.7	3.5 ± 0.3	1.1 ± 0.1	278.3 ± 4.5	281.7 ± 7.7
FW	6	605.7 ± 8.0**	216.4 ± 4.1***	2.4 ± 0.3*	0.7 ± 0.1*	175.4 ± 3.0***	142.3 ± 2.2**
Environmental water							
SW	3	1,012.8 ± 5.4	545.0 ± 5.6	6.8 ± 2.4	56.5 ± 0.3	572.7 ± 1.5	<0.0
FW	3	17.0 ± 4.0	7.2 ± 2.5	0.6 ± 0.0	0.8 ± 0.2	11.2 ± 0.9	<0.0

Values are presented as means ± s.e.m. Statistically significant differences between SW and FW individuals are shown with asterisks (*p < 0.05, **p < 0.01, ***p < 0.001).

**TABLE 2:** Compositions of plasma and urine collected from SW- and 5% SW-acclimated red stingray in Experiment 2

	N	Osmolality (mOsm/kg)	Na^+^ (mM)	Ca^2+^ (mM)	Mg^2+^ (mM)	Cl^−^ (mM)	Urea (mM)
Plasma							
SW	5	1,045.9 ± 5.0	363.3 ± 10.7	4.1 ± 0.7	0.9 ± 0.2	300.8 ± 10.8	324.1 ± 12.8
5% SW	6	623.5 ± 20.7***	210.3 ± 6.9***	2.4 ± 0.2	0.5 ± 0.1	173.1 ± 6.4***	194.0 ± 7.8***
Urine							
SW	5	1,040.0 ± 18.6	344.7 ± 96.4	21.7 ± 7.1	264.6 ± 55.8^†^	81.9 ± 18.9^†††^	84.0 ± 32.5^†^
5% SW	6	172.8 ± 13.4***^†††^	32.3 ± 4.3^†††^	1.1 ± 0.2^††^	0.7 ± 0.2*	20.2 ± 4.8*^†††^	91.9 ± 9.0^†††^
Environmental water							
SW	5	1,054.5 ± 5.4	552.1 ± 4.0	8.7 ± 2.0	52.9 ± 0.4	551.3 ± 2.4	<1.0
5% SW	6	53.4 ± 3.7	22.6 ± 2.0	1.4 ± 0.2	2.6 ± 0.2	26.0 ± 2.1	<1.0

Values are presented as means ± s.e.m. Comparisons within the same parameters were corrected by Holm method. Statistically significant differences between SW, and 5% SW, individuals, and between plasma and urine are shown with asterisks and daggers, respectively (*, †*p* < 0.05, ††*p* < 0.01, ***, †††*p* < 0.001).

**FIGURE 1:**
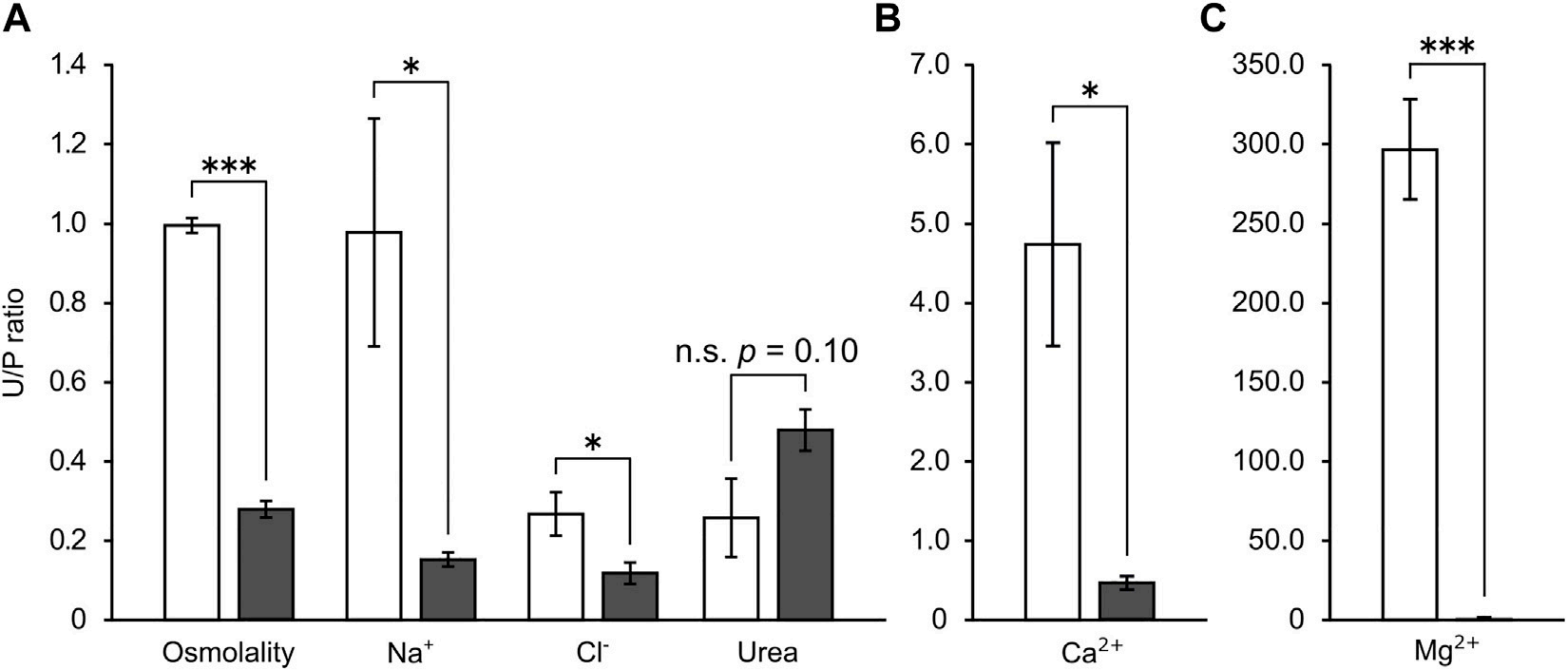
The urine/plasma (U/P) ratios of major osmolytes **(A)** and divalent ions **(B,C)** in the stingray. Open and filled bar represent values from SW- and 5% SW-acclimated stingrays, respectively. Asterisks indicate statistically significant differences between SW- and 5% SW-acclimated stingrays (*p < 0.05, ***p < 0.001).

In SW control red stingray, the urine osmolality and Na^+^ level were nearly identical to those of plasma, while urine Cl^−^ and urea levels were significantly lower than those in plasma. In contrast, Ca^2+^ tended to be higher in urine than plasma (*p* = 0.069 after correction by Holm method) and Mg^2+^ was significantly higher in urine than in plasma (approximately 5.3 and 294.0 times concentrated in urine for Ca^2+^ and Mg^2+^, respectively). Following the acclimation to 5% SW, urine levels of osmolality, Mg^2+^, and Cl^−^ were significantly decreased (Table 2). Urine levels of Na^+^ were 10 times lower in individuals acclimated to 5% SW than SW control individuals, but no significant difference was observed due to the high variation in SW control individuals. Overall, the decreases in osmolality and ion concentrations were greater in urine than in plasma. In consequence, the urine/plasma (U/P) ratios of osmolality and ions were significantly lower in 5% SW-acclimated individuals than in SW control individuals (SW versus 5% SW groups; osmolality, 0.99 ± 0.02 vs. 0.28 ± 0.02; Na^+^, 0.98 ± 0.29 vs. 0.15 ± 0.02; Cl^−^, 0.27 ± 0.05 vs. 0.12 ± 0.03; Ca^2+^, 4.75 ± 1.28 vs. 0.46 ± 0.08; Mg^2+^, 297.6 ± 31.54 vs. 1.67 ± 0.56) (Figure 1). However, urea concentration in urine was similar between SW control and 5% SW-acclimated individuals, and thus, the U/P ratio of urea slightly but insignificantly increased in 5% SW-acclimated individuals (SW versus 5% SW groups; 0.26 ± 0.10 vs. 0.48 ± 0.05) (Figure 1A).

Currently, values of GFR and UFR of conscious red stingrays in SW and FW environments are not available. Therefore, with the GFRs and UFRs reported in euryhaline Atlantic stingray (Janech et al., 2006), we estimated the reabsorption rate of Na^+^, Cl^−^, and urea (Table 3). In Janech et al. (2006), GFR and UFR values were determined in SW- and 50% SW-acclimated stingrays. Although the salinity of diluted media was greatly different between the previous study (50% SW) and the current investigation (5% SW), we considered that borrowing the GFR and UFR values from the study of Atlantic stingrays is insightful on estimating the changes in the urine production capacity under different salinities as the GFR and UFR were significantly increased following the transfer of stingrays from SW to 50% SW (Janech et al., 2006). In the SW control group of the red stingrays, urea is reabsorbed to a slightly greater extent than NaCl, whereas the reabsorption rate of Na^+^ and Cl^−^ was greater than that of urea in the FW-acclimated stingrays. Compared to the SW control, the reabsorption rates of Na^+^, Cl^−^, and urea were significantly increased in the 5% SW-acclimated stingrays by 2.2-, 1.9-, and 1.4-fold, respectively (Table 3).

**TABLE 3:** Estimated reabsorption rate of major osmolytes in the red stingrays.

		Reabsorption rate (μmol/hour/kg)		
	N	Na^+^	Cl^−^	Urea
SW	5	1,070.1 ± 122.1	1,069.3 ± 30.0	1,156.0 ± 52.0
5% SW	6	2,347.0 ± 70.3***	1982.8 ± 79.0***	1,662.1 ± 133.2**

Values are presented as means ± s. e.m. Asterisks indicate statistically significant differences between SW- and 5% SW-acclimated stingrays (**p < 0.01, ***p < 0.001). For the calculation, reported values of GFR [SW; 3.8 (ml/hour/kg), 5% SW; 12.4 (ml//hour/kg)] and UFR [SW; 0.9 (ml/hour/kg), 5% SW; 8.1 (ml//hour/kg)] were adopted from Atlantic stingray acclimated to natural and 50% diluted harbor water, respectively (Janech et al., 2006).

### Expression profiles of transporters involved in NaCl and urea reabsorption

To search for the candidate genes that are responsible for the enhanced reabsorption of NaCl and urea in low-salinity environments, the transcriptomes of the kidney were examined in SW control and FW-acclimated individuals. The analysis of the sample correlation matrix on the overall expression profiles showed a clear clustering between SW control and FW-acclimated stingrays (Supplementary Figure S2). By using the selection criteria of DEGs described in our previous study (Imaseki et al., 2019), we identified 131 annotated genes upregulated in FW after the application of dual cut-off [false discovery rate (FDR) < 0.05 and log2 fold change (logFC) > 1.5 of count per million (CPM) value] (Supplementary Table S2). Among them, we focused on solute carrier (*slc*) family genes as they play pivotal roles in osmolyte transport in osmoregulatory epithelia (Hediger et al., 2004). The following eight *slc* genes were found to be upregulated: facilitated glucose transporter member 1 (*slc2a1*, *glut1*, 5.3-fold), sodium-coupled monocarboxylate transporter 1 (*slc5a8*, *smct1*, 5.7-fold), sodium-sulfate cotransporter (*slc13a1*, *nas1*, 12.8-fold), monocarboxylate transporter 4 (*slc16a3*, *mot4*, 5.9-fold), sulfate transporter (*slc26a2*, *dtdst*, 7.8-fold), ammonium transporter Rh type B (*slc42a2*, *rhbg*, 3.3-fold), large neutral amino acids transporter small subunit 4 (*slc43a2*, *lat4*, 2.9-fold), and sodium-dependent lysophosphatidylcholine symporter 1-B (*slc59a1*, *nls1b*, 7.7-fold) (Supplementary Table S2). However, no candidate genes encoding known transporters or channels for Cl^−^ or urea transport were found among the DEGs. To further explore the transcriptome, we subsequently extracted expression data of the following *slc* family genes known to be involved in NaCl and urea transport: *slc5* (sodium glucose cotransporter), *slc9* (sodium/proton exchanger), *slc12* (electroneutral cation-coupled chloride cotransporter), and *slc14* (urea transporter) (Table 4). In addition, expression data of chloride channel (*clc*), epithelial sodium channel (*enac*), *nka*, and FXYD domain-containing ion transport regulator [*fxyd*; often regarded as the third NKA subunit (Pirkmajer and Chibalin, 2019)] were also extracted (Table 4). Among the *slc* genes involved in NaCl transport, *nkcc2* (*slc12a1*) has the highest Transcripts Per Kilobase Million (TPM) values, and the values in FW-acclimated stingray were 1.9-fold higher than those of SW control individuals. The TPM values of NKA alpha subunit 1 (*nkaα1*) and *fxyd3* were comparable to those of *nkcc2*, and 2.5-fold increase in the TPM values of *nkaα1* was observed in FW-acclimated individuals. The TPM values of *nkaβ1* subunit, *fxyd2*, and *fxyd3* were increased 3.7-, 8.1-, and 1.3-fold in FW-acclimated stingrays, respectively. The expression of the *ncc* (*slc12a3*) was decreased in the FW-acclimated stingrays, and the TPM values are much lower than those of *nkcc2*.

**TABLE 4:** Expression of genes putatively linked to NaCl and urea reabsorption in the transcriptomic analysis.

			TPM value					
Transcript contig ID	Annotation	Gene symbol	SW1	SW2	SW3	FW1	FW2	FW3
DN56997_c6_g2	Solute carrier family 5 (sodium/glucose cotransporter), member 1 [*Leucoraja erinacea*]	*slc5a1*, *sglt1*	3.1	4.1	1.3	5.6	4.7	24.8
DN48755_c5_g1	Sodium/glucose cotransporter 2 isoform X2 [*Chiloscyllium plagiosum*]	*slc5a2*, *sglt*2	23.8	49.7	50.0	73.7	97.3	93.2
DN57379_c1_g2	Sodium/myo-inositol cotransporter-like isoform X1 [*Amblyraja radiata*]	*slc5a3*, *smit1*	71.4	53.2	56.0	61.4	69.8	31.7
DN56128_c7_g1	High-affinity choline transporter 1-like [*Amblyraja radiata*]	*slc5a7*, *cht*	0.9	1.0	4.6	1.5	1.9	4.4
DN47922_c8_g2	Sodium-coupled monocarboxylate transporter 1 isoform X1 [*Amblyraja radiata*]	*slc5a8*, *smct1*	7.0	5.2	4.2	18.9	25.4	48.8
DN51220_c0_g1	Sodium/glucose cotransporter 4 [*Amblyraja radiata*]	*slc5a9*, *sglt4*	25.0	31.0	39.8	19.4	15.5	12.2
DN55339_c6_g1	Sodium/glucose cotransporter 5 isoform X1 [*Carcharodon carcharias*]	*slc5a10*, *sglt5*	2.2	1.4	1.3	1.7	2.6	2.0
DN57283_c3_g2	Sodium/myo-inositol cotransporter 2-like isoform X1 [*Amblyraja radiata*]	*slc5a11*, *smit2*	139.2	194.1	77.4	201.1	180.4	102.6
DN57206_c4_g2	Sodium-coupled monocarboxylate transporter 2 isoform X2 [*Chiloscyllium plagiosum*]	*slc5a12*, *smct2*	18.1	8.2	36.6	6.3	24.3	20.4
DN51815_c0_g1	Sodium/hydrogen exchanger 1 [*Amblyraja radiata*]	*slc9a1*, *nhe1*	2.0	2.0	2.1	3.5	3.1	2.9
DN46781_c0_g1	Sodium/hydrogen exchanger 2-like [*Amblyraja radiata*]	*slc9a2*, *nhe2*	2.9	3.8	5.3	2.1	2.7	3.9
DN52182_c12_g3	Na^+^/H^+^ exchanger type 3 [*Hypanus sabinus*]	*slc9a3*, *nhe3*	9.4	8.9	19.4	4.3	4.9	5.9
DN54402_c2_g1	Sodium/hydrogen exchanger 7 isoform X1 [*Carcharodon carcharias*]	*slc9a7, nhe7*	1.8	0.9	0.8	0.8	0.6	0.3
DN47838_c4_g1	Sodium/hydrogen exchanger 8 isoform X1 [*Amblyraja radiata*]	*slc9a8*, *nhe8*	9.7	10.0	9.3	5.3	6.3	6.6
DN46008_c0_g1	Sodium/hydrogen exchanger 9-like isoform X1 [*Carcharodon carcharias*]	*slc9a9*, *nhe9*	2.6	3.1	4.6	2.1	1.6	4.0
DN56595_c4_g1	Solute carrier family 12 member 1*	***slc12a1*, *nkcc2***	367.1	297.5	496.9	777.2	720.7	667.9
DN48109_c9_g1	Na^+^:K^+:^2Cl- cotransporter 1 [*Himantura signifer*]	*slc12a2*, *nkcc1*	6.1	7.6	5.5	4.5	5.3	6.6
DN50058_c2_g1	Solute carrier family 12 member 3 [*Amblyraja radiata*]	*slc12a3*, *ncc*	20.2	13.8	31.5	4.3	10.7	1.4
DN53154_c3_g2	PREDICTED: solute carrier family 12 member 6 isoform X1 [*Latimeria chalumnae*]	*slc12a6*, *kcc3*	8.3	7.1	7.1	7.3	6.4	6.7
DN53606_c0_g1	Solute carrier family 12 member 7 isoform X2 [*Amblyraja radiata*]	*slc12a7*, *kcc4*	4.7	8.6	8.2	8.5	8.4	6.0
DN51351_c0_g1	Solute carrier family 12 member 8 isoform X1 [*Carcharodon carcharias*]	*slc12a8*, *ccc9*	2.5	6.3	5.3	3.2	3.8	3.4
DN53601_c4_g5	Solute carrier family 12 member 9-like isoform X1 [*Amblyraja radiata*]	*slc12a9*, *ccc6*	1.4	0.9	0.8	0.8	0.7	0.9
DN52195_c5_g1	Urea transporter*	***slc14a1***, ***ut***	16.1	19.8	24.0	21.1	23.7	31.6
DN54090_c2_g1	H (^+^)/Cl (^-^) exchange transporter 3 isoform X4 [*Chiloscyllium plagiosum*]	*clc-3*	11.9	10.8	10.6	8.6	10.5	8.4
DN48716_c0_g1	H (^+^)/Cl (^-^) exchange transporter 5 isoform X1 [*Scyliorhinus canicula*]	*clc-5*	6.3	5.7	6.3	6.0	5.4	7.5
DN52817_c0_g2	Chloride transport protein 6 [*Amblyraja radiata*]	*clc-6*	6.5	8.0	5.8	4.6	4.5	4.8
DN46657_c0_g1	H (^+^)/Cl (^-^) exchange transporter 7 isoform X1 [*Amblyraja radiata*]	*clc-7*	9.7	9.6	8.2	12.8	8.6	6.2
DN57075_c9_g2	Chloride channel protein ClC-Kb-like [*Carcharodon carcharias*]	clc-kb**	7.6	9.3	11.4	8.9	6.1	7.1
DN56714_c7_g2	Chloride channel K isoform X1 [*Carcharodon carcharias*]	clc-kb**	39.1	53.3	54.0	62.4	80.4	97.1
DN51921_c1_g2	Amiloride-sensitive sodium channel subunit alpha-like [*Scyliorhinus canicula*]	*enacα*	12.1	16.5	19.6	17.2	22.7	30.3
DN54588_c5_g2	Amiloride-sensitive sodium channel subunit beta [*Amblyraja radiata*]	*enacβ*	6.4	7.3	2.4	9.5	8.0	6.5
DN54241_c13_g1	**Na** ^**+**^ **/K** ^**+**^ transporting subunit alpha 1*	***nkaα1***	306.9	345.1	383.2	815.8	847.1	1,009.2
DN45503_c0_g1	Na^+^/K^+^-ATPase alpha-subunit 2 [*Himantura signifer*]	*nkaα2*	0.8	1.2	1.3	0.4	0.6	0.9
DN53748_c3_g2	RecName: Full = Sodium/potassium-transporting ATPase subunit beta-1; AltName: Full = Sodium/potassium-dependent ATPase subunit beta-1 [*Tetronarce californica*]	*nkaβ1*	133.3	134.9	144.9	417.7	486.2	604.8
DN52334_c7_g1	Hypothetical protein [*Chiloscyllium punctatum*]	nkaβ3**	4.4	6.4	7.2	3.9	2.2	7.5
DN51046_c4_g1	Protein ATP1B4 [*Scyliorhinus canicula*]	*nkaβ4*	9.8	9.2	5.5	5.6	6.2	5.9
DN46337_c6_g3	FXYD domain-containing ion transport regulator 6-like [*Rhincodon typus*]	fxyd2**	8.6	19.2	40.5	108.3	63.3	387.0
DN58223_c7_g1	FXYD domain-containing ion transport regulator 3-like [*Chiloscyllium plagiosum*]	*fxyd3*	365.1	321.1	439.3	496.4	466.4	548.3
DN55291_c9_g1	FXYD domain-containing ion transport regulator 6 [*Carcharodon carcharias*]	fxyd6**	0.1	0.2	0.1	1.2	1.3	2.8

Expression levels were shown in Transcripts Per Kilobase Million (TPM). *Previously cloned sequence (Aburatani et al., 2020). **Orthology was checked by ORTHOSCOPE (Inoue and Satoh, 2019).

To obtain the molecular information on membrane transporters for divalent ions, we also extracted the expression data of the following gene families: *slc8* (sodium/calcium exchanger), *slc13* (sodium sulfate cotransporter), *slc26* (sulfate/anion exchanger), *slc41* (sodium/magnesium exchanger), Cyclin M divalent cation transport mediator (*cnnm*), Transient receptor potential cation channel subfamily M (*trpm*), Transient receptor potential cation channel subfamily V (*trpv*), and ATPase plasma membrane calcium transporting (*pmca*) (Table 5). Among the genes with the TPM values higher than 20, we observed that the expression levels of *slc13a3*, *slc13a4*, *slc26a6*, and *slc41a1* were decreased, whereas those of *slc26a1* and *trpm6* were increased.

**TABLE 5:** Expression of genes putatively linked to divalent ion reabsorption in the transcriptomic analysis.

			TPM value					
Transcript contig ID	Annotation	Gene symbol	SW1	SW2	SW3	FW1	FW2	FW3
DN41214_c0_g1	Sodium/calcium exchanger 1-like isoform X4 [*Carcharodon carcharias*]	*slc8a1*, *ncx1*	1.2	0.2	0	0	0.8	0.5
DN131733_c0_g1	Sodium/calcium exchanger 3-like isoform X1 [*Carcharodon carcharias*]	*slc8a3*, *ncx3*	0.3	0.4	0	0.1	0.6	0.2
DN57715_c13_g2	Solute carrier family 13 member 1 [*Gopherus evgoodei*]	*slc13a1*, *nas1*	0.4	0.5	0.3	6.1	4.5	3.3
DN55252_c11_g1	Solute carrier family 13 member 2 [*Amblyraja radiata*]	*slc13a2*, *nadc1*	5.8	9.9	3.1	10.7	10.3	17.2
DN57644_c12_g1	Solute carrier family 13 member 3 [*Amblyraja radiata*]	*slc13a3*, *nadc3*	412.1	304.4	309.2	136.2	98.1	51.7
DN47621_c0_g1	Solute carrier family 13 member 4 [*Chiloscyllium plagiosum*]	*slc13a4*, *sut1*	98.7	83.7	208.6	14.1	12.0	8.0
DN55553_c8_g1	Sulfate transporter-like [*Amblyraja radiata*]	*slc26a1*, *sat*-1**	32.4	6.6	12.3	183.3	124.7	89.5
DN39385_c0_g1	Sulfate transporter [*Amblyraja radiata*]	*slc26a2*, dtdst**	0.2	0.2	0.4	0.1	0.2	0.1
DN55312_c0_g2	Prestin isoform X1 [*Amblyraja radiata*]	*slc26a5*, *pres*	6.2	5.6	3.6	8.4	10.3	5.8
DN51950_c3_g1	Solute carrier family 26 member 6 [*Chiloscyllium plagiosum*]	*slc26a6*	76.6	117.8	111.5	7.6	7.5	2.4
DN53138_c4_g1	Solute carrier family 26 member 9 [*Amblyraja radiata*]	*slc26a9*	5.2	5.0	4.4	2.7	1.9	0.7
DN46515_c10_g1	Solute carrier family 26 member 10 [*Carcharodon carcharias*]	*slc26a10*	0.8	0.5	0.6	0.2	0.1	0.1
DN56056_c5_g2	Sodium-independent sulfate anion transporter [*Rhincodon typus*]	*slc26a11*	5.9	9.3	5.3	7.6	8.6	6.3
DN53519_c12_g1	Solute carrier family 41 member 1 [*Amblyraja radiata*]	slc41a1**	131.0	88.8	70.4	31.5	27.9	14.4
DN49105_c7_g2	Solute carrier family 41 member 1 isoform X1 [*Carcharodon carcharias*]	slc41a3**	1.0	1.4	0.6	0.3	0.5	1.6
DN46730_c7_g1	Metal transporter CNNM1 [*Amblyraja radiata*]	*cnnm1*	4.3	5.1	6.0	2.1	5.2	3.9
DN46730_c8_g2	Metal transporter CNNM2 isoform X1 [*Amblyraja radiata*]	*cnnm2*	7.3	5.8	8.5	7.0	6.3	5.7
DN46612_c11_g1	Metal transporter CNNM3-like isoform X1 [*Amblyraja radiata*]	*cnnm3*	1.0	1.3	1.1	1.4	1.1	1.8
DN53777_c5_g1	Transient receptor potential cation channel subfamily M member 6 [*Amblyraja radiata*]	*trpm6*	8.1	11.0	7.7	21.4	20.7	11.7
DN52096_c10_g1	Transient receptor potential cation channel subfamily M member 7 isoform X3 [*Amblyraja radiata*]	*trpm7*	13.5	18.0	12.1	9.6	12.3	7.7
DN47960_c6_g1	Transient receptor potential cation channel subfamily V member 4 [*Carcharodon carcharias*]	*trpv4*	0.6	0.5	0.3	0.4	0.3	0.6
DN133231_c0_g1	Transient receptor potential cation channel subfamily V member 5-like isoform X2 [*Amblyraja radiata*]	*trpv5*	0	0	0	0	0	0.2
DN47186_c0_g1	Transient receptor potential cation channel subfamily V member 6 [*Scyliorhinus canicula*]	*trpv6*	0.4	0.2	1.0	3.3	4.9	5.4
DN46788_c0_g3	Plasma membrane calcium-transporting ATPase 1 isoform X2 [*Amblyraja radiata*]	*pmca1*	4.6	4.5	6.3	3.8	4.4	5.0
DN52137_c2_g2	Plasma membrane calcium-transporting ATPase 2 isoform X3 [*Chiloscyllium plagiosum*]	*pmca2*	10.4	3.9	13.8	2.3	2.1	0.8

Expression levels were shown in TPM. **Orthology was checked by ORTHOSCOPE (Inoue and Satoh, 2019).

### Changes in gene expression of *nkcc2*, *nkaα1*, *ut*, and *ncc*

**FIGURE 2:**
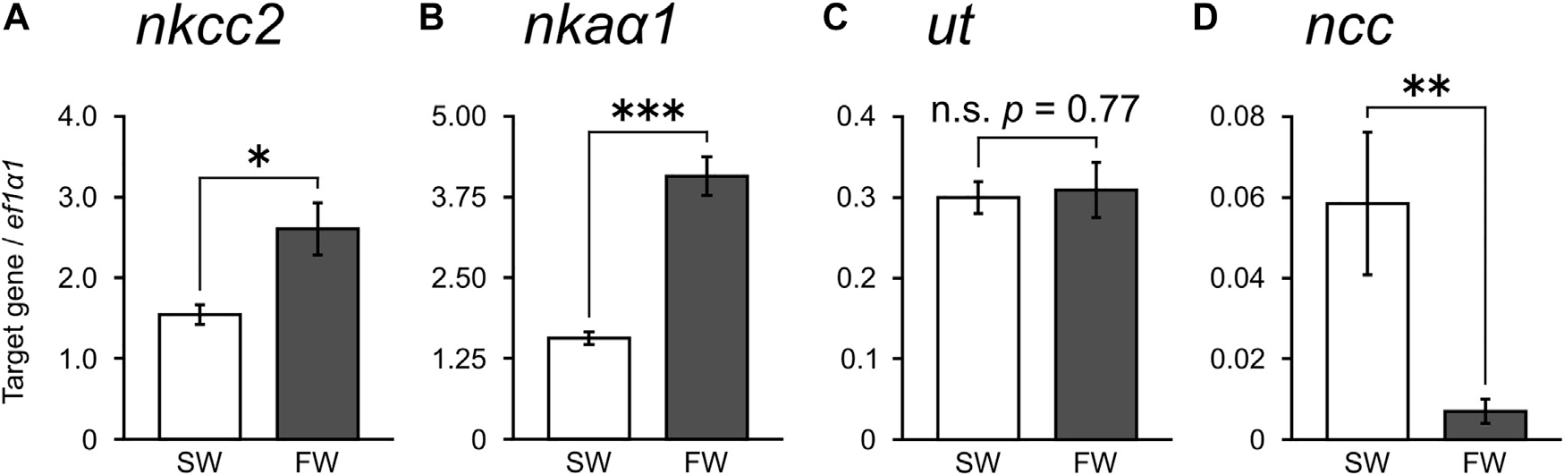
Expression of transporter mRNAs in the kidney measured by real-time qPCR. mRNAs for *nkcc2* **(A)**, *nkaα1* **(B)**, *ut* **(C)**, and *ncc* **(D)** were quantified and normalized against those of elongation factor 1α1 (*ef1α1*). *N* = 6 in each group. Asterisks indicate statistically significant differences between SW control and FW-acclimated stingrays (*p < 0.05, **p < 0.01, ***p < 0.001).

Using qPCR, we subsequently investigated the expression levels of *nkcc2*, *nkaα1*, urea transporter (*ut*), and *ncc* that have been reported to be important for NaCl and urea reabsorption of the kidney in cartilaginous fishes (Hyodo et al., 2014; Imaseki et al., 2019; Aburatani et al., 2020). The expression levels of *nkcc2* and *nkaα1* mRNAs in the kidney of FW-acclimated stingray were significantly higher than those of SW control individuals (1.7- and 2.6-fold, respectively; Figures 2A, B). However, no significant difference was observed in the expression of *ut* mRNA between SW control and FW-acclimated stingrays (Figure 2C). The expression of *ncc* mRNA was significantly lower in the FW-acclimated stingrays than in the SW control individuals (Figure 2D). These expression patterns examined by qPCR were consistent with those of RNA-seq analyses. No difference was observed in gene expression levels between sexes both in SW control (*nkcc2*, *p* = 0.16; *nkaα1*, *p* = 0.71; *ut*, *p* = 0.41; *ncc*, *p* = 0.69) and FW-acclimated stingrays (*nkcc2*, *p* = 0.92; *nkaα1*, *p* = 0.20; *ut*, *p* = 0.50; *ncc*, *p* = 0.54).

### Distribution and expression levels of *nkcc2*, *nkaα1*, *ut*, and *ncc* mRNAs in the stingray nephron

**FIGURE 3:**
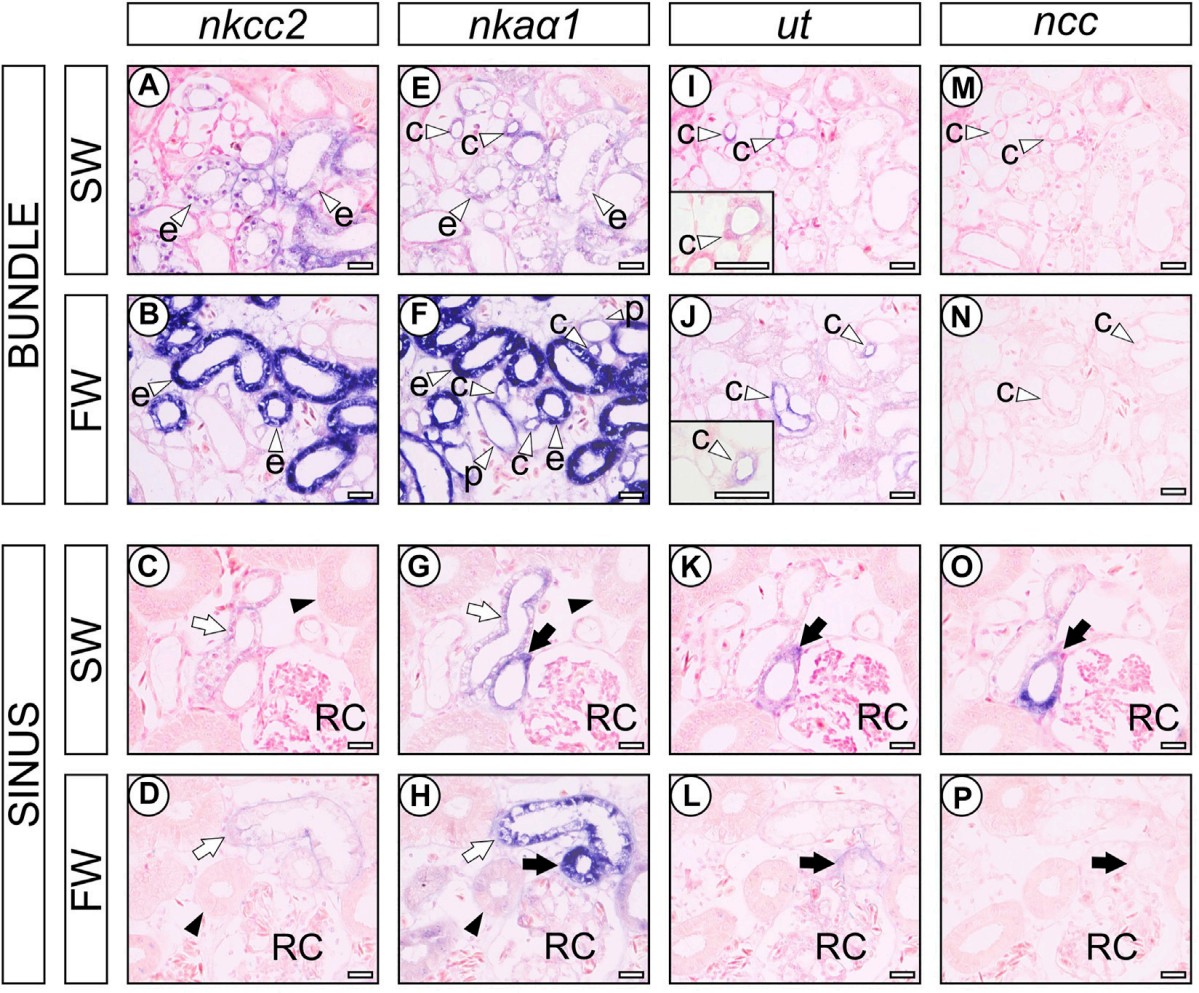
*In situ* hybridization analysis of transporter genes in the nephrons of SW control and FW-acclimated stingrays. *nkcc2* **(A–D)**, *nkaα1* **(E–H)**, *ut* **(I-L)**, and *ncc* **(M-P)** in SW control **(A,C,E,G,I,K,M,O)** and FW-acclimated **(B,D,F,H,J,L,N,P)** stingray. Open arrowheads indicate the PI (labeled with “p”), EDT (labeled with “e”), and CT (labeled with “c”) in the bundle zone **(A,B,E,F,I,J,M,N)**. Filled arrowheads indicate the PII **(C,D,G,H)**. Open and filled arrows indicate the ascending and descending LDT, respectively **(C,D,G,H,K,L,O,P)**. RC, renal corpuscle. Bars, 50 μm. Note that 1) the *nkcc2* mRNA was intensely expressed in the EDT of FW-acclimated stingrays, but no difference was observed in LDT between SW control and FW-acclimated stingrays, and 2) concomitantly, prominent *nkaα1* mRNA signals were observed in the EDT of FW-acclimated stingrays.

Intense *nkcc2* mRNA signals were observed in the largest columnar cells of early distal tubule (EDT) in the bundle zone (open arrowhead labeled “e” in Figures 3A,B), and weak signals for *nkcc2* mRNA were continued to the ascending late distal tubules (LDT) in the sinus zone (open arrows in Figures 3C, D), which are relatively smaller diameter than the proximal tubules II (PII) (filled arrowhead in Figures 3C, D). The signal intensity of *nkcc2* mRNA in the EDT of FW-acclimated individuals was considerably stronger than that of SW control individuals, while no obvious difference was found in the LDT between SW control and FW-acclimated individuals. *nkaα1* mRNA signals were found in a wide range of renal tubules, and the strongest signals were observed in the EDT in the bundle zone (Figures 3E, F). In the bundle zone, the signal intensities of *nkaα1* mRNA were higher in the proximal tubule I (PI) (open arrowheads labeled “p” in Figure 3F), collecting tubule (tubules “c” in Figures 3E, F), and EDT (tubules “e” in Figures 3E, F) of FW-acclimated individuals than those of SW control. The increases in signal intensity of *nkaα1* mRNA were the most prominent in the EDT of the bundle zone, and this coincided well with the increased *nkcc2* mRNA signals in the FW-acclimated individuals. In the sinus zone, *nkaα1* mRNA signals were increased in the ascending and descending LDT (open and filled arrows, respectively) and PII segment (filled arrowhead) in the FW-acclimated individuals (Figures 3G, H). Of note, we controlled the time for chromogenic reaction of *nkcc2* and *nkaα1* mRNA signals in order to compare signal intensities between SW and FW individuals. If the reaction time was extended, *nkcc2* and *nkaα1* mRNA signals were noticeable in the EDT even in SW control individuals, but differences in signal intensity were diminished between SW control and FW-acclimated individuals.

The *ut* mRNA signals were found in the collecting tubule (CT) and preceding transitional region between LDT and CT in the vicinity of renal corpuscles both in SW control and FW-acclimated individuals. The collecting tubules were characterized by the cuboidal epithelial cells located in the center of the tubular bundle and the signal intensity of *ut* mRNA was stronger in FW-acclimated individuals (open arrowhead labeled “c” in Figures 3I, J). In contrast, *ut* mRNA signals in the transitional region between LDT and CT in sinus zone were decreased in FW-acclimated individuals (filled arrow in Figures 3K, L). In SW control stingray, *ncc* mRNA signal was detected in the *ut* mRNA-positive tubule of the sinus zone (filled arrow in Figures 3K, O), but not in the collecting tubule of the bundle zone (open arrowhead labeled “c” in Figures 3I, M). However, *ncc* mRNA signals were almost undetectable in FW-acclimated individuals (Figures 3N, P).

**FIGURE 4:**
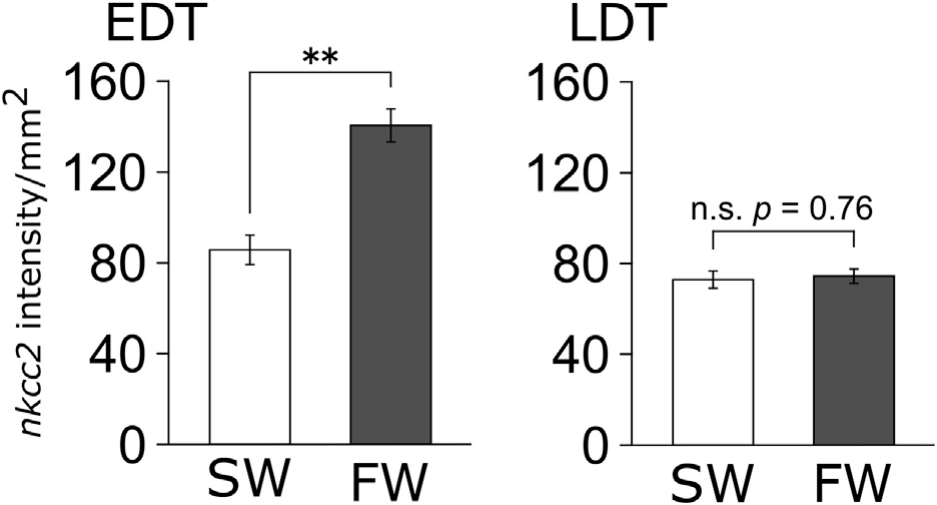
Signal intensity of *nkcc2* mRNA expressions in EDT and LDT. The signals of *nkcc2* mRNA expression in the EDT (left panel) and LDT (right panel) were quantified with ImageJ. Open and filled columns represent SW control and FW-acclimated individuals, respectively. *N* = 4 in each group. Statistically significant difference between SW and FW is shown with asterisks (**p < 0.01).

Because the results of *in situ* hybridization indicate segment-specific upregulation of *nkcc2*, we analyzed the signal intensity of *nkcc2* mRNA expressions in each segment, namely, EDT and LDT (Figure 4). As with the observations of *in situ* hybridization images, the signal intensity of *nkcc2* mRNA was significantly increased in the EDT inside the tubular bundles. This was not observed in the LDT of the sinus zone.

## Discussion

In the present study, we found that the red stingray is similar to other euryhaline elasmobranchs as the red stingray maintained high levels of plasma NaCl and urea that make up an osmolality over 600 mOsm in low-salinity environments. Euryhaline elasmobranchs produce dilute urine to cope with a massive water influx caused by the steep osmotic gradient between body fluid and low-salinity environments. Consistent with the previous findings in sawfish (Smith, 1931), Atlantic stingray (Janech and Piermarini, 2002), and bull shark (Imaseki et al., 2019), red stingray excreted dilute urine following the acclimation to diluted environments. Our results further implied that the red stingray seems to possess a higher capacity to produce dilute urine than bull shark, for which physiological and molecular investigation of kidney function was conducted following the acclimation to FW in a similar manner to the present study (Imaseki et al., 2019). When we compared the U/P ratio of each parameter, the U/P ratios of osmolality, Na^+^, and Cl^−^ were significantly decreased following the acclimation to 5% SW in red stingray. In FW-acclimated bull sharks, the Na^+^ and Cl^−^ concentrations in the urine were 5- and 7-fold higher than those of 5% SW-acclimated red stingrays (Imaseki et al., 2019), leading to our hypothesis that the mechanisms of diluted urine production are different between bull shark and red stingray.

In the present study, GFR and UFR values were not available in conscious red stingrays acclimated to SW and low-salinity environments. Therefore, we borrowed the GFR and UFR values reported in the Atlantic stingray reared in ambient SW and 50% SW to estimate differences in the solute reabsorption rate between the SW control and 5% SW-acclimated red stingrays. The estimated values in Table 3 indicate that the transfer of red stingray from SW to 5% SW caused significant (1.4- to 2.2-fold) increases in reabsorption of NaCl and urea from the glomerular filtrate, suggesting that the red stingrays enhanced reabsorption of both NaCl and urea in low-salinity environments. However, it should be noted that our reported solute reabsorption rates in 5% SW-acclimated stingray may be underestimated. Since red stingrays in FW or 5% SW have larger osmotic difference between body fluid and holding water than the case of Atlantic stingray, the red stingrays must have experienced a greater influx of water. Therefore, it is reasonable to assume that the red stingray in FW or 5% SW should have a higher GFR and UFR.

### Anatomical and molecular characteristics of the red stingray nephron to produce dilute urine

RNA-seq and subsequent qPCR analyses revealed that *nkcc2* (*slc12a1*) is the most abundantly expressed gene among candidate solute carrier (*slc*) family proteins involved in the NaCl reabsorption. The expression of *nkcc2* mRNA was significantly increased following the acclimation to FW environments. NKCC2 is an apically localized membrane protein contributing to active reabsorption of NaCl in the distal segment of FW teleost nephron (Takvam et al., 2021) and the thick ascending limb of the loop of Henle in the mammalian nephron (Bazúa-Valenti et al., 2016). In these diluting segments, NKCC2 reabsorbs NaCl from the glomerular filtrate in coordination with the basolateral NKA. Our data also indicated higher mRNA levels of *nkaα1*, *nkaβ1*, *fxyd2*, and *fxyd3* in FW-acclimated individuals, implying that the apical NKCC2 and basolateral NKA system was enhanced for producing dilute urine in the FW.

We previously found that the red stingray possesses a highly convoluted and elongated EDT segment, where NKCC2 and NKA are coexpressed for reabsorbing NaCl from the glomerular filtrate (Aburatani et al., 2020). In the present study, *in situ* hybridization data demonstrated that *nkaα1* and *nkcc2* mRNA levels were upregulated in the EDT of the stingray nephron following the acclimation to FW. Although *nkcc2* mRNA was also found in LDT, the upregulation was only observed in the EDT. On the other hand, in bull shark, no increase was observed in the expression levels of *nkcc2* mRNA in the EDT following the transfer from SW to FW, implying that the difference in the expression levels of *nkcc2* mRNA causes the higher urinary concentrations of Na^+^ and Cl^−^ in the FW-acclimated bull sharks than the 5% SW-acclimated red stingrays. Low levels of Cl^−^ in the urine ware also reported in sawfish *Pristis microdon* and Atlantic stingray *Hypanus sabinus* captured in FW environments (Smith, 1931; Janech and Piermarini, 2002). Although molecular and anatomical investigations are needed in sawfish and Atlantic stingray, we hypothesize that the remarkable ability of batoid nephrons to produce dilute urine is attributable to the enhanced expression of *nkcc2* and *nka* mRNAs in the well-developed EDT.

**FIGURE 5:**
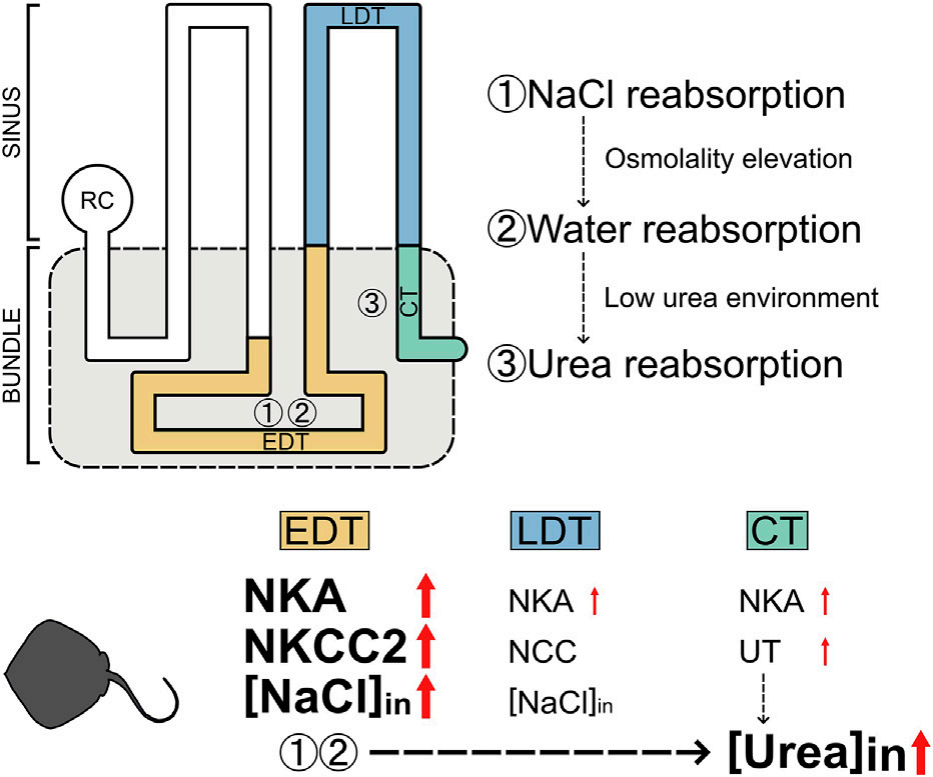
Schematic summary for the enhancement of NaCl and urea reabsorption in the kidneys of red stingray following acclimation to low-salinity environment. [NaCl]_in_ and [Urea]_in_ indicate reabsorptions of NaCl and urea, respectively.

In addition to NaCl, the estimated reabsorption rate for urea was also increased in the stingray acclimated to low-salinity environments. The mechanism for the higher urea reabsorption in low-salinity environments is probably explainable with the urea reabsorption model proposed in cartilaginous fish (Figure 5; Hyodo et al., 2014). This model is composed of three steps: 1) active reabsorption of NaCl, 2) passive reabsorption of water, and 3) facilitative urea reabsorption in the bundle zone. The first step is active transport of NaCl from primary urine into the interstitial space in the microenvironment wrapped by the impermeable peritubular sheath (Lacy and Reale, 1986). NKCC2 and NKA expressed in the EDT are responsible for this step, resulting in elevation of interstitial osmolality inside the peritubular sheath. The elevated interstitial osmolality leads to water reabsorption via the aquaporin-expressing segment such as the EDT (Cutler et al., 2022), which creates a low-urea interstitial fluid inside the peritubular sheath. After these two steps, urea is left in processed filtrate and being concentrated. In the final step, urea is reabsorbed through the facilitative UT expressed in the collecting tubule (Hyodo et al., 2004; Kakumura et al., 2015; Aburatani et al., 2020) using the concentration gradient of urea as a driving force. Therefore, it is highly probable that the enhanced NaCl reabsorption in the EDT (the first step) consequently caused a greater amount of urea reabsorption in the kidney of FW (or 5% SW)-acclimated stingray (Figure 5).

In bull shark, NCC appeared to be a key molecule contributing to the successful FW acclimation (Imaseki et al., 2019). Approximately 10-fold increase was detected in the expression of *ncc* mRNA, and the *ncc* mRNA signals were remarkably abundant in the LDT (the fourth loop), which concomitantly expressed high level of *nka* mRNA. The NCC and NKA system in the FW bull shark is thought to increase the NaCl reabsorption in the sinus zone. In the stingray, on the other hand, *ncc* expression is limited to the transitional portion between LDT and CT as with houndshark (Imaseki et al., 2019). Furthermore, the expression levels of *ncc* were decreased following the FW acclimation. These results suggest that the mechanisms to produce dilute urine are different between red stingray and bull shark. Since the LDT is located outside of the peritubular sheath, the enhanced NaCl reabsorption in the LDT cannot affect the interstitial osmolality in the microenvironment wrapped by the peritubular sheath, which could be the reason why the estimated amount of reabsorbed urea from the primary urine is lower in FW-acclimated bull shark (Imaseki et al., 2019) than 5% SW-acclimated red stingray.

In FW-acclimated red stingrays, upregulation of *nkaα1* mRNA signals was observed not only in the EDT, but also in most segments in the bundle zone. Relatively high *nkaα1* expression was found in the PI and CT in the bundle zone of the kidney from FW-acclimated red stingray, suggesting that enhancement of active solute transports is not limited to the EDT for elevating the interstitial osmolality. Although currently an apically located transporter/channel, which functions in coordination with NKA, remains to be clarified in the PI and CT of FW-acclimated red stingray, reabsorption of any solutes could result in the further elevation of interstitial osmolality.

It has been well-documented that excretion of divalent ions is a crucial renal function of SW fishes, including cartilaginous fishes (Beyenbach, 2000; McDonald, 2007), as SW contains higher divalent ions than the body fluid. Indeed, the U/P ratio of divalent cations (in particular Mg^2+^) was higher than that of monovalent cations in the SW control red stingray. Following the transfer to 5% SW, the U/P ratios of Ca^2+^ and Mg^2+^ decreased by 90.3 and 99.4%, respectively, indicating that excretory functions of divalent ions were strongly suppressed as the divalent ion influxes stopped in low-salinity environments. The genes encoding *slc13a3*, *13a4*, *26a6*, and *41a1* were abundantly expressed in the kidney of SW control stingrays, while FW transfer decreased the expression of those genes, suggesting that they may contribute to the excretion of divalent ions into the urine. In holocephalan elephant fish (*Callorhinchus milii*), the PII within the sinus zone expressed SLC26A1 and SLC26A6 at basolateral and apical membranes, respectively, and they were related to sulfate secretion (Hasegawa et al., 2016), supporting the putative role of SLC26A6 in divalent ion excretion in the red stingray nephron. In red stingray, however, the expression of *slc26a1* was upregulated in FW-acclimated individuals. Further studies are needed to investigate the mechanisms regulating divalent ion homeostasis in SW and low-salinity environments in batoids.

### Perspectives: Evolution of euryhalinity in elasmobranchs

The subclass Elasmobranchii is composed of Selachii and Batoidea. Several species in Batoidea are known to be euryhaline, while only bull shark and their close relatives such as *Glyphis* are known euryhaline selachians. In addition, Grant et al. (2019) recently categorized 19 batoids as estuarine generalists, which suggests that they may have certain abilities to survive in wide ranges of salinity. The categorized batoid species are phylogenetically diverse, including Dasyatidae, Pristidae, Rajidae, Rhinidae, and Rhinopteridae, while no selachians satisfied their criteria. These facts suggest that batoids have some intrinsic quality to accommodate low-salinity environments. Our previous investigation showed that in the batoid-type nephron, the EDT is more convoluted than that in selachians (Aburatani et al., 2020). Furthermore, we demonstrated that the red stingray has remarkable capacity to produce dilute urine, which is partly related to the enhanced expression of *nkcc2* and *nka* mRNAs in the highly convoluted EDT. These characteristics of the stingray nephron indicate that the batoid-type nephron could be advantageous for expanding their habitat to a wide range of salinity environments. Our data showed that red stingray and bull shark use different renal mechanisms for the acclimation to low-salinity environments, supporting the notion that batoids and selachians have acquired euryhalinity through the separate evolutionary trajectories (Ballantyne and Fraser, 2012). Further comparative studies on renal morphology and physiological responses to different environmental conditions in other euryhaline elasmobranchs are necessary to deepen our understanding of the osmoregulatory strategies in cartilaginous fishes.

## Data Availability

The datasets presented in this study can be found in online repositories. The names of the repository/repositories and accession number(s) can be found in the article/Supplementary Material.
