# Supplementary material for: Molecular and morphological investigations on the renal mechanisms enabling euryhalinity of red stingray Hemitrygon akajei
Source: Front Physiol. 2022 Aug 9;13:953665. doi: 10.3389/fphys.2022.953665 (PMC9396271; doi:10.3389/fphys.2022.953665)
Supplement: Supplementary file 1 [file DataSheet2.PDF]

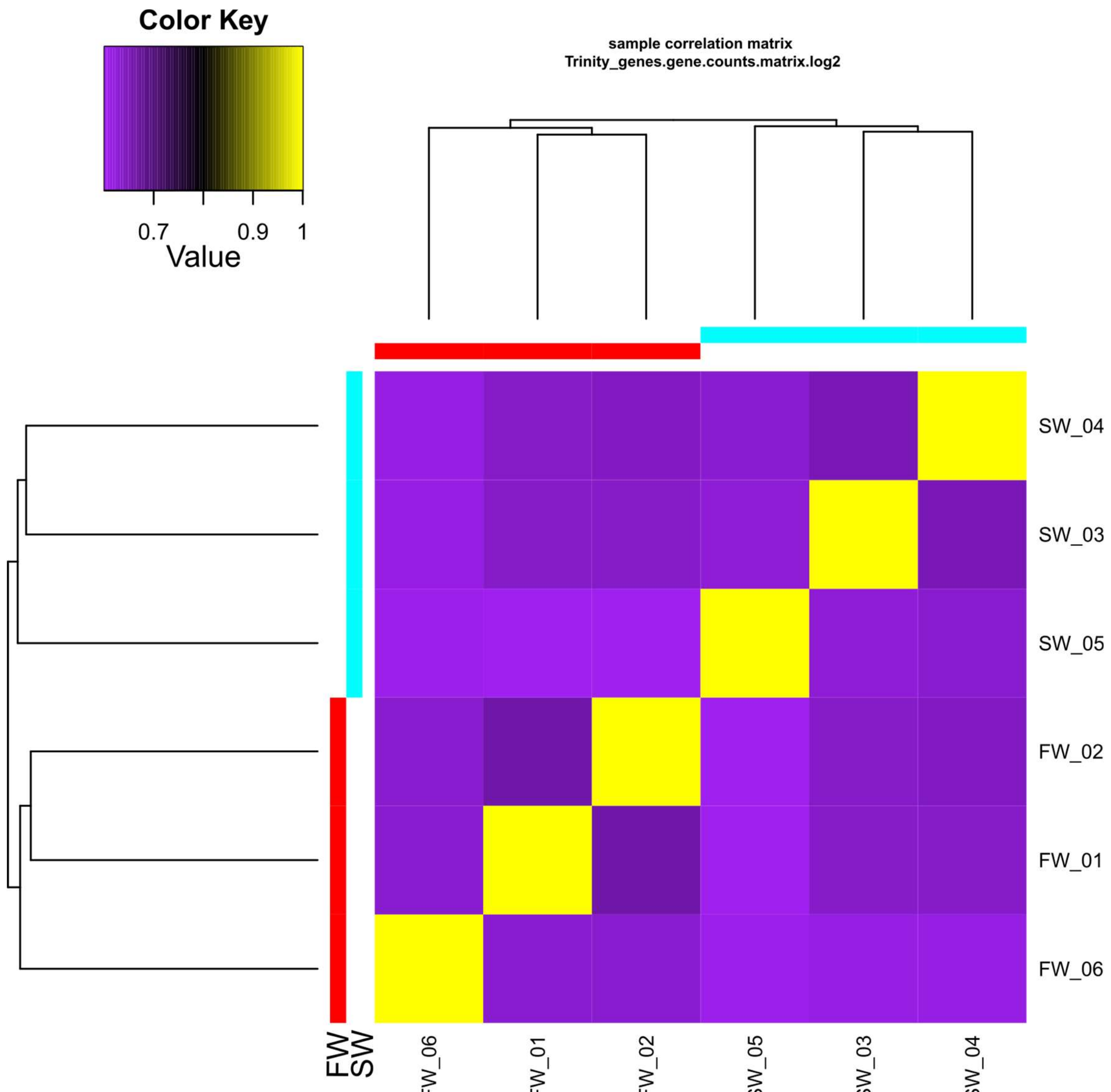

Fig S2. Analysis of sample correlation matrix based on expression profile in the red stingray kidney.
