# Supplementary material for: Molecular and morphological investigations on the renal mechanisms enabling euryhalinity of red stingray Hemitrygon akajei
Source: Front Physiol. 2022 Aug 9;13:953665. doi: 10.3389/fphys.2022.953665 (PMC9396271; doi:10.3389/fphys.2022.953665)
Supplement: Supplementary file 3 [file DataSheet1.pdf]

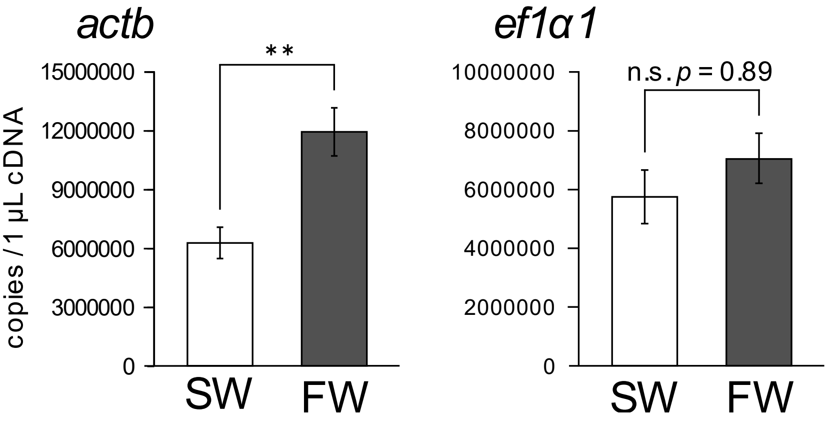

Fig. S1. Expression levels of house-keeping genes. Asterisks indicate statistically-significant difference between SW control and FW-acclimated stingrays (** $P < 0.01$).
